# Network insights into childhood obesity: unveiling methylated-differentially expressed genes and pathways through integrative bioinformatics analysis

**DOI:** 10.1530/EC-25-0049

**Published:** 2025-06-06

**Authors:** Felipe Mateus Pellenz, Guilherme Coutinho Kullmann Duarte, Giovanna Câmara Giudicelli, Thayne Woycinck Kowalski, Taís Silveira Assmann, Daisy Crispim

**Affiliations:** ^1^ Endocrine Division, Hospital de Clínicas de Porto Alegre, Porto Alegre, Rio Grande do Sul, Brazil; ^2^ Universidade Federal do Rio Grande do Sul, Faculty of Medicine, Department of Internal Medicine, Graduate Program in Medical Sciences: Endocrinology, Porto Alegre, Rio Grande do Sul, Brazil; ^3^ Bioinformatics Core, Hospital de Clínicas de Porto Alegre, Porto Alegre, Rio Grande do Sul, Brazil; ^4^ Universidade Federal do Rio Grande do Sul, Department of Genetics, Graduate Program in Genetics and Molecular Biology, Porto Alegre, Rio Grande do Sul, Brazil; ^5^ Universidade Federal do Rio Grande do Sul, Graduate Program in Medical Sciences, Porto Alegre, Rio Grande do Sul, Brazil; ^6^ Medical Genetics Service, Hospital de Clínicas de Porto Alegre, Porto Alegre, Rio Grande do Sul, Brazil

**Keywords:** childhood obesity, differentially methylated genes, methylation-regulated differentially expressed genes (MeDEGs), bioinformatics, systems biology

## Abstract

**Background:**

Childhood obesity, a global epidemic with profound impacts on physical and psychological health, remains a complex challenge with elusive underlying mechanisms. This study aimed to unravel the epigenetic landscape of this disease by identifying methylated-differentially expressed genes (MeDEGs) in childhood obesity through integrated bioinformatics approaches.

**Methods:**

Expression profiling (GSE9624) and methylation profiling (GSE25301, GSE27860, and GSE57484) datasets containing data on children with obesity (cases) and eutrophic children (control group) were obtained from the Gene Expression Omnibus (GEO) repository. Differentially expressed genes (DEGs) and differentially methylated genes (DMGs) between the groups were identified using GEO2R. MeDEGs were identified by superimposing the lists of DEGs and DMGs. The protein–protein interaction (PPI) network was constructed using the STRING database and analyzed using Cytoscape. Topological and modular PPI network analyses were carried out using the CytoHubba and MCODE plugins, respectively. Functional enrichment analyses were performed based on Gene Ontology terms and KEGG pathways.

**Results:**

A total of 70 MeDEGs were identified, including 45 hypomethylated high-expression and 25 hypermethylated low-expression genes. The PPI network highlighted three hub-bottleneck genes (*CCL5*, *STAT1*, and *GATA3*) and two functional modules. Overall, the 70 MeDEGs were associated with KEGG pathways related to cellular differentiation, inflammation, chemokine signaling, lipid and glucose metabolism, insulin resistance, and apoptosis.

**Conclusion:**

This study, employing integrative bioinformatics approaches, provides insights into the methylation-mediated mechanisms contributing to childhood obesity, advancing our understanding of this multifaceted chronic disease.

## Introduction

Childhood obesity is a chronic metabolic disease characterized by excessive body weight, resulting from a long-term imbalance between energy intake and energy expenditure (1). Alarmingly, the global prevalence of this disease is increasing, making it a global burden (1). Children with obesity are five times likely to maintain their adiposity profile into adulthood compared to lean children (2, 3), significantly increasing their risk of developing future diseases, including type 2 diabetes mellitus, cardiovascular disease, and cancer, which can lead to premature death (4, 5, 6).

Childhood obesity is a multifactorial disease influenced by a complex interplay among endocrine, environmental, sociocultural, behavioral, genetic, and epigenetic factors (4, 7, 8). Recent evidence indicates that up to 1,100 independent genetic loci with small-to-modest effects are linked to obesity pathogenesis (8). A meta-analysis of 27 studies involving 7,928 children and adolescents with overweight/obesity revealed that polymorphisms in 24 genetic loci are significantly associated with changes in body mass index (BMI) and body composition (8). Moreover, twin studies suggest that the genetic heritability of obesity may be as high as 47–90% (9).

Epigenetic factors play a crucial role in regulating the intricate interplay between various environmental clues and gene expression, without altering the DNA sequence (10). Among these factors, DNA methylation is one of the most extensively studied in several diseases, including childhood obesity (7, 11). DNA methylation involves the addition of a methyl group to the C-5′ position of a cytosine residue by DNA methyltransferases (DNMTs) in CpG dinucleotides, also known as CpG islands (7, 12, 13, 14). In addition, non-CpG methylation is also detected in humans. Notably, unmethylated CpG islands in gene promoters are typically associated with transcriptionally active genes, whereas methylated CpG islands in gene promoters are linked to transcriptional repression (7, 12, 14). Research on DNA methylation in children is of special importance, as evidence suggests that methylation patterns established early in life tend to persist over time (7). An Epigenome-Wide Association Study (EWAS) including 903 mother–child pairs from the Boston Birth Cohort identified 123 differentially methylated CpG islands associated with childhood overweight and obesity (15). In addition, the study by Patel *et al.* (16), which included 31 children, revealed 3,133 differentially methylated CpG islands between children with overweight/obesity and lean controls. Among these, 792 CpG islands were hypermethylated, while 1,239 were hypomethylated (16). These findings consistently demonstrate an association between childhood obesity and changes in methylation at both the gene and genome-wide levels (6).

In recent years, high-throughput profiling arrays have been extensively utilized to screen changes in gene expression and DNA methylation patterns in various diseases (17, 18). Moreover, integrating bioinformatics analyses of these profiling arrays through systems biology approaches offers promising tools for enhancing our understanding of diseases (17, 18, 19). While most studies typically separate analyses regarding differentially expressed genes (DEGs) and differentially methylated genes (DMGs) (17, 18, 19), there is an emerging trend toward integrating gene expression and DNA methylation datasets. This integration provides a holistic approach, yielding methylation-regulated differentially expressed genes (MeDEGs), which can contribute significantly to a deeper understanding of childhood obesity pathogenesis rather than relying on disconnected analyses (17, 19, 20). Therefore, our study was aimed to utilize a systems biology-based integrative analysis to identify MeDEGs in children with obesity compared to controls. In addition, we sought to unravel the molecular mechanisms and metabolic pathways associated with these MeDEGs, contributing to a nuanced understanding of the complex events involved in the development of childhood obesity.

## Materials and methods

### Microarray data

**Figure 1:**
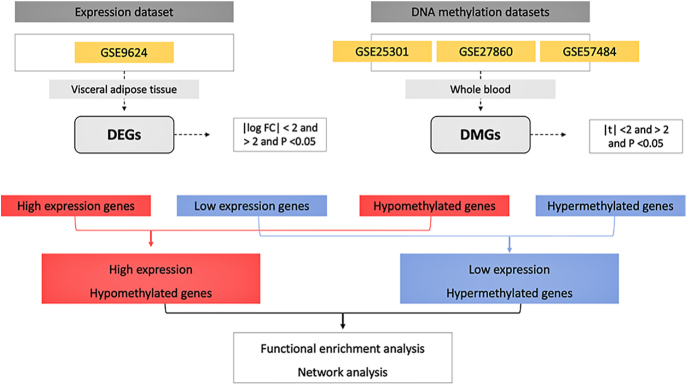
Flowchart of the identification of methylation-regulated differentially expressed genes (MeDEGs) in childhood obesity. DEG, differentially expressed genes; DMG, differentially methylated genes.

We conducted a search for gene expression and DNA methylation profiling datasets related to childhood obesity in the Gene Expression Omnibus (GEO; https://www.ncbi.nlm.nih.gov/geo), a public repository of various high-throughput functional genomics data, including microarrays and next-generation sequencing (NGS). For this study, our GEO search strategy involved the following terms: ‘pediatric obesity’ [MeSH terms] AND ‘*Homo sapiens*’ [Organism] AND (‘DNA methylation’ OR ‘Expression profiling’ [Filter]). This search strategy resulted in the identification of four microarray datasets, as depicted in Fig. 1. These datasets, which were included in this study, comprised one gene expression dataset (GSE9624) and three DNA methylation datasets (GSE25301, GSE27860, and GSE57484), all involving various samples of patients with childhood obesity and lean children.

The GSE9624 dataset (GLP570; Affymetrix Human Genome U133 Plus 2.0 Array) analyzed gene expression in the visceral adipose tissue (VAT) from six lean children (five male/one female; mean age ± SD: 8.2 ± 0.6; mean BMI ± SD: 16.8 ± 0.6) and five male children with obesity (mean age: 10.2 ± 0.6; mean BMI: 28.1 ± 0.7) (21). Regarding the DNA methylation datasets, the GSE25301 (GPL8490; Illumina HumanMethylation27 BeadChip) analyzed isolated leukocytes from seven male lean children (mean age: 15.9 ± 1.4; mean BMI: 17.0 ± 0.7) and seven male children with obesity (mean age: 15.8 ± 1.0; mean BMI: 39.0 ± 1.7) (22); GSE27860 (GPL8490; Illumina HumanMethylation27 BeadChip) included analyses of whole blood from 24 female controls (mean age: 10.5 ± 0.4; mean BMI: 17.2 ± 0.8) and 23 female cases with childhood obesity (mean age: 11.0 ± 0.6; mean BMI 27.0 ± 1.0) (23); and GSE57484 (GPL8490; Illumina HumanMethylation27 BeadChip) analyzed whole blood of 11 male preadolescents without obesity (mean age: 10.3 ± 0.3; mean BMI z-score: −0.71 ± 0.11) and 11 male children with obesity (mean age: 10.8 ± 0.6; mean BMI z-score: 1.60 ± 0.2) (24). The Illumina HumanMethylation27 BeadChip platform includes methylome analyses of >27,000 CpG islands in promoters of >14,500 genes (25).

### Data processing

GEO2R (https://www.ncbi.nlm.nih.gov/geo/geo2r/) is an intuitive web tool that enables researchers to compare two or more groups of samples within a GEO Series. In this study, we utilized GEO2R to analyze the included datasets and identify both DMGs and DEGs in samples from children with obesity compared to lean children. The Limma v.3.26.8 package (26) for R v.3.2.3 (https://www.r-project.org/) was employed to normalize DMGs corresponding to multiple probe IDs, estimating the average value of these probes as the representative expression level of a given gene. It is important to note that all gene identifiers were mapped according to the HUGO Gene Nomenclature Committee (HGNC) (27).

DEGs were defined based on an absolute log2-fold change (log FC) <2.0 or >2.0 with a significance level of *P* < 0.05. For DMGs, the cutoff points were set as |*t*| > 2 and *P* < 0.05. Subsequently, the datasets were integrated based on their gene expression and methylation profiles using Venn diagrams on the InteractiveVenn website (28). This integration allowed us to identify genes that were both upregulated and hypomethylated, indicating hypomethylated high-expression genes. Similarly, genes that were both downregulated and hypermethylated were identified as hypermethylated low-expression genes. Importantly, MeDEGs were identified by considering both hypomethylated high-expression and hypomethylated low-expression genes.

### Protein–protein interaction network

The protein–protein interaction (PPI) network of MeDEGs associated with childhood obesity was systematically assembled using the Search Tool for the Retrieval of Interacting Genes (STRING v.11.5; https://string-db.org/) database (29). For this purpose, interactions with a moderate interaction score >0.4 and a significance level of *P* < 0.05 were considered statistically significant. Results obtained from STRING were then imported into Cytoscape v.3.10.0 (30) for comprehensive network analyses and visualization. Importantly, no shell interactors were introduced to the network in STRING, ensuring that the constructed PPI network reflects direct interactions among the identified MeDEGs, thereby enhancing the accuracy and specificity of the network analysis.

### Systems biology approach to analyze the PPI network based on MeDEGs

Complex biological interactions can be analyzed and represented as a graph, illustrating the mechanistic relationships among proteins from a given network (31). A network can be represented by nodes that are interconnected by edges, depicting the relationships among the nodes (31). In this study, the nodes are genes and edges are lines that symbolize the interactions between the genes, forming a PPI network.

Two centrality measures, degree and betweenness, were applied to assess the relevance of each node within the network. Degree is an algorithm that measures the number of connections of each node, with highly interconnected nodes referred to as hubs, which tend to be important elements of control within the network (32). Moreover, betweenness estimates the number of minimum non-redundant paths between two nodes that cross a specific node (33). Nodes with high betweenness are called bottlenecks as they usually act as major intersections between modules in different networks (33). In this study, genes in the top 10% of degree and betweenness distributions, with at least two interactions, were considered hubs and bottlenecks, respectively (34). Hub and bottleneck genes were identified using the CytoHubba plugin version 0.1 (35) for Cytoscape.

Functional modules within the PPI network were identified using cluster analysis with the Molecular Complex Detection (MCODE) version 2.0.0 plugin (36) for Cytoscape, with a cutoff point set at a number of nodes >5.0. The MCODE algorithm facilitated the recognition of highly interconnected regions in the PPI network, serving as potential functional complexes where genes and proteins within modules tend to share common biological functions (36, 37).

### Functional enrichment analysis

The Kyoto Encyclopedia of Genes and Genomes (KEGG) is a comprehensive resource encompassing databases on genomes, biological pathways, diseases, and chemical substances (38). Gene Ontology (GO) terms are widely used to determine biological attributes related to genes, gene products, and sequences, including biological processes (BP), molecular function (MF), and cell components (CC) (39). KEGG pathways were sourced from the pathDIP version 5 database (40). GO term analyses were performed using STRING version 11.5. Statistical tests for functional enrichment analyses were based on the hypergeometric test, and *P* values were corrected for multiple tests using the Benjamini–Hochberg procedure, yielding false discovery rate (FDR)-adjusted *P* values (*q*-values). GO terms and KEGG pathways associated with *q*-values <0.05 were considered statistically significantly enriched. Graphics were constructed using Plotly chart studio (https://chart-studio.plotly.com/feed/#/).

## Results

### Identification of DMGs and DEGs in childhood obesity

**Figure 2:**
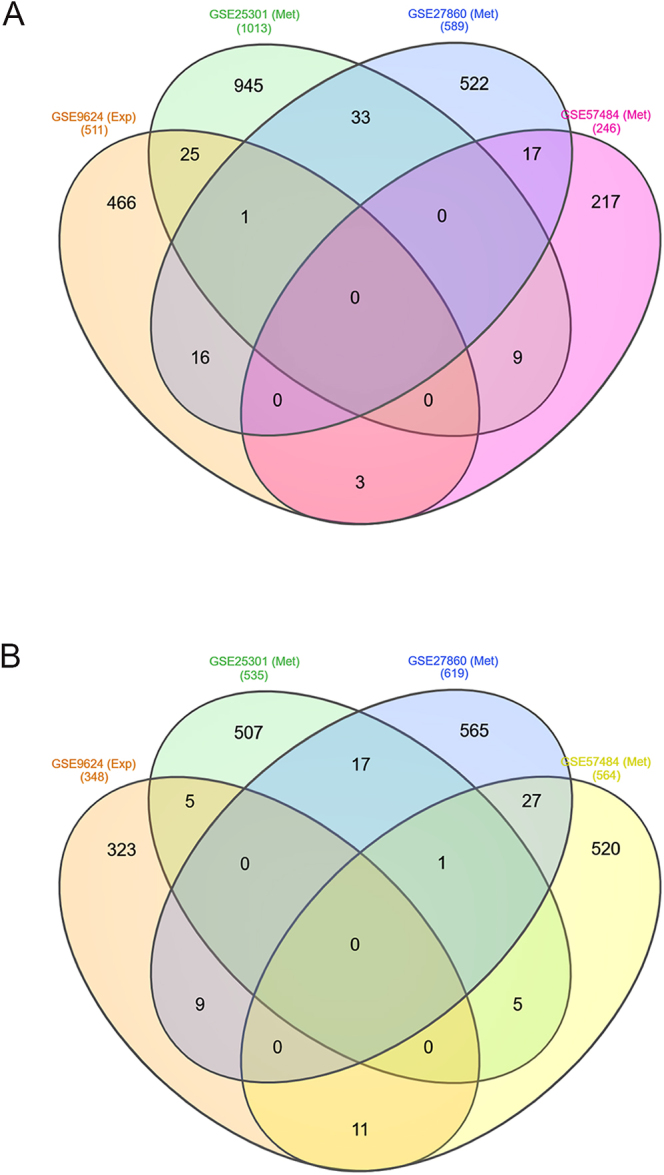
Identification of methylation-regulated differentially expressed genes (MeDEGs) in childhood obesity by superimposition of gene expression (Exp) dataset (GSE9624) and DNA methylation (Met) datasets (GSE25301, GSE27860, and GSE57484). (A) Hypomethylated high-expression genes. (B) Hypermethylated low-expression genes.

Figure 1 illustrates the flowchart of the strategy of analyses used in this study. For the analysis of DEGs in VAT biopsies, we identified 511 upregulated and 348 downregulated genes in the gene expression dataset (GSE9624). Regarding DMGs in whole blood, after combining the three datasets (GSE25301, GSE27860, and GSE57484), we identified 1,667 hypermethylated and 1,788 hypomethylated genes. Moreover, upon combining all four datasets, we identified 45 hypomethylated high-expression (Fig. 2A) and 25 hypermethylated low-expression genes (Fig. 2B) shared by at least one DEG/DMG dataset, totalizing 70 MeDEGs. At the time of manuscript preparation, no paired datasets were available, nor was there a whole-blood transcriptome dataset for childhood obesity or a methylome dataset for VAT biopsies in childhood obesity. Consequently, our findings may have differed if analyzing paired datasets.

### PPI network construction, identification of hub and bottleneck genes, and module analysis

**Figure 3:**
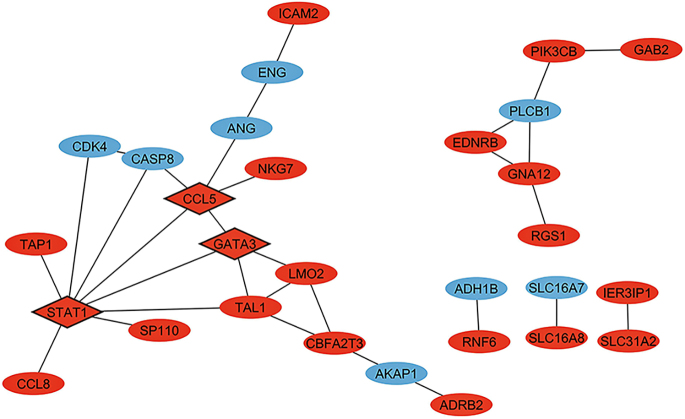
PPI network formed by the 29 MeDEGs. Red nodes represent hypomethylated high-expression genes in childhood obesity, while blue nodes represent hypermethylated low-expression genes. Diamond nodes represent hub-bottleneck genes identified through network topological analysis. Nodes represent the proteins that are encoded by each gene and edges represent PPIs.

The PPI network, constructed using the STRING database, comprises 29 nodes representing MeDEGs and 31 connecting edges (lines), which reflect PPIs (Fig. 3). Nodes represent proteins encoded by each gene, while edges represent PPIs. Among the initially considered 70 MeDEGs, 41 lacked interconnections within the network and were therefore excluded from subsequent analyses. Considering the 29 MeDEGs depicted in the PPI network, eight were hypermethylated low-expression genes, while 21 were hypomethylated high-expression genes. This categorization highlights the distinct methylation and expression patterns characterizing the genes within the network, underscoring the complexity of their regulatory relationships and potential functional implications in the context of childhood obesity.

**Figure 4:**
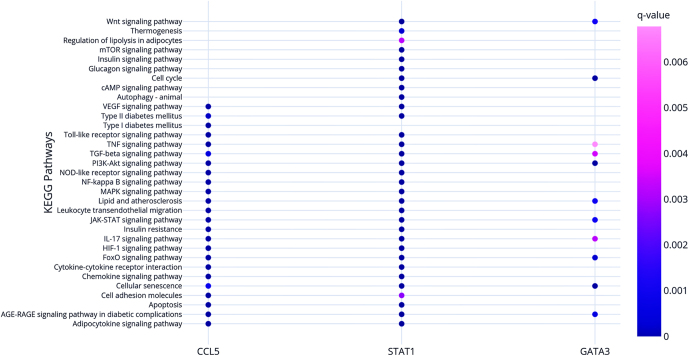
Significantly enriched KEGG pathways in which each hub-bottleneck gene participates. The color of the circle represents statistical significance according to *q*-values.

Hub and bottleneck genes were assessed using degree and betweenness centrality measures, respectively. The topological analyses of the PPI network are described in Supplementary Table 1 (see section on Supplementary materials given at the end of the article). The three most connected nodes were considered as hub-bottleneck genes: signal transducer and activator of transcription 1 (*STAT1*), C–C motif chemokine ligand 5 (*CCL5*), and GATA-binding protein 3 (*GATA3*), which could play a critical role in childhood obesity. These genes participate in KEGG pathways such as cellular senescence, apoptosis, PI3K-Akt, TNF, JAK-STAT, NF-kappa B, toll-like receptor, NOD-like receptor, and FoxO signaling pathways (Fig. 4). Supplementary Table 2 shows the entire list of enriched KEGG pathways for the three hub-bottleneck genes. Regarding GO terms, these genes enriched molecular functions related to CCR5 chemokine receptor signaling, transcription coactivator binding, and cytokine receptor binding. Biological processes enriched by these genes were related to cellular responses to TNF and cell differentiation.

**Figure 5:**
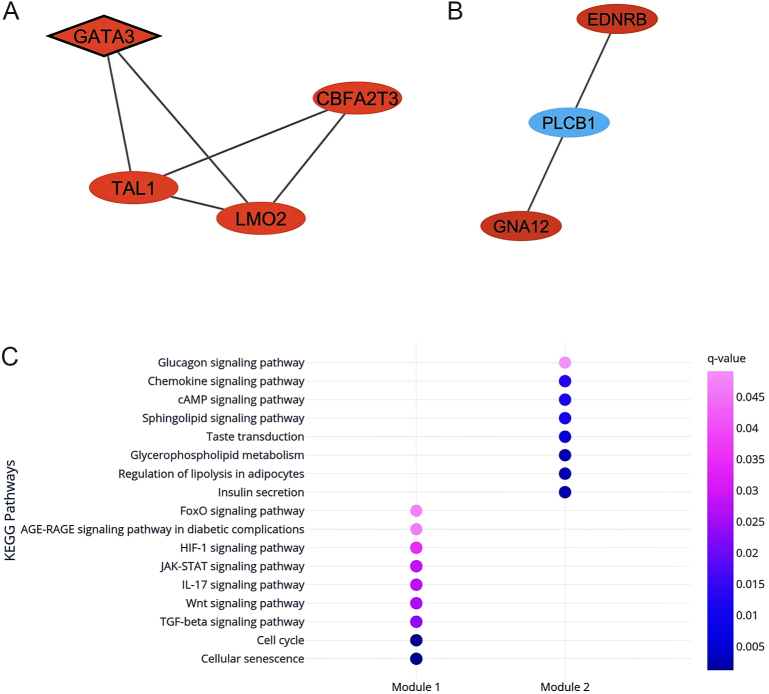
Module analysis showing the genes present in each functional module. (A) Module 1: red nodes represent hypomethylated high-expression genes in childhood obesity, while diamond nodes represent hub-bottleneck genes identified in the PPI network analysis. (B) Module 2: red nodes represent hypomethylated high-expression genes, while blue nodes represent hypermethylated low-expression genes. (C) Functional enrichment analysis demonstrating the enriched KEGG pathways of each functional module; the color of the circle represents statistical significance according to *q*-values.

Module analyses enabled the demonstration of groups of genes that likely act together in a PPI network. Our analyses revealed two modules in the network: Module 1 (Fig. 5A) had a module score of 3.3, while Module 2 (Fig. 5B) scored 3.0. Module 1 consisted of four nodes (*CBFA2T3*, *GATA3*, *LMO2*, and *TAL1*) and five edges, while Module 2 included three nodes (*EDNRB*, *GNA12*, and *PLCB1*) and three edges. Notably, *GATA3*, a hub-bottleneck gene, was a part of Module 1. In addition, Module 1 was enriched in several KEGG pathways, such as cellular senescence, JAK-STAT, HIF-1, and Wnt signaling pathways (Fig. 5C), while Module 2-enriched pathways were related to inflammation and regulation of lipid and glucose metabolism (Fig. 5C). The complete lists of KEGG pathways for each module are provided in Supplementary Table 3. Regarding GO terms, the genes included in Module 1 were enriched in biological processes related to myeloid leukocyte differentiation, while Module 2 did not present statistically significant enriched GO terms.

### Functional enrichment analyses of MeDEGs

Supplementary Table 4A provides a comprehensive overview of the 190 enriched KEGG pathways for the 70 MeDEGs. These genes also enriched GO terms related to immune system processes. The 45 hypomethylated high-expression MeDEGs were specifically involved in immune system-related biological processes. Regarding molecular functions, these MeDEGs were also associated with RNA polymerase II-specific DNA-binding transcription factor. In contrast, the hypermethylated low-expression MeDEGs did not show significant enrichment in GO terms.

Supplementary Table 4B presents the 128 KEGG pathways significantly enriched exclusively by the 45 hypomethylated high-expression MeDEGs. These genes are involved in leukocyte differentiation and maturation, chemokine signaling and function, lipid and glucose metabolism, and apoptosis. Supplementary Table 4C describes the 149 KEGG pathways significantly enriched exclusively by the 25 hypermethylated low-expression MeDEGs. Notably, this set of genes is mainly involved in cellular senescence, cell homeostasis, longevity, chemokine signaling, insulin resistance (IR), leukocyte activation, and lipid and glucose metabolism.

## Discussion

The present study contributes to the understanding of MeDEGs putatively involved with childhood obesity by integrating large-scale datasets of DMGs and DEGs. Childhood obesity is recognized as an important risk factor to various diseases in adulthood (7, 21). Some studies have shown a consistent association between childhood obesity and DNA methylation at both gene and genome-wide scales (7, 21, 22, 23, 24). However, interpreting these findings is complicated by the inherent heterogeneity present in the data (7). Combining information from large datasets of DMGs and DEGs through advanced bioinformatics methods allows for the identification of key genes that are concomitantly influenced by both differential methylation and gene expression (19). Here, by applying integrated bioinformatics approaches, we identified 70 MeDEGs, shedding light on potential key regulators of childhood obesity.

The categorization into hypomethylated high-expression and hypermethylated low-expression genes provides a more detailed understanding of the nuanced gene expression patterns associated with childhood obesity. Functional enrichment analyses revealed that the identified MeDEGs may play a crucial role in regulating the immune system. Furthermore, the discovery of three hub-bottleneck genes (*CCL5*, *STAT1*, and *GATA3*) offers intriguing insights into the pathways associated with childhood obesity, including insulin, immune, and inflammatory-related pathways. Interestingly, these three hub-bottlenecks were among the hypomethylated high-expression genes.

*CCL5*, encoding the protein of same name, plays a crucial role in inflammation by binding to its receptor (CCR5), thereby facilitating the recruitment of immune cells to inflammatory sites. Its impact extends across a variety of cells related to the innate and adaptive immune systems (41, 42). Interestingly, a cross-sectional study involving children with obesity revealed a significant increase in *CCL5* expression compared to eutrophic children (43). Consistently, decreased methylation in the promoter region of the *CCL5* gene was observed in peripheral blood mononuclear cells (PBMCs) of preadolescents with obesity, aligning with elevated *CCL5* expression compared to healthy controls (44). Moreover, a previous study reported elevated serum levels of CCL5 in patients with type 2 diabetes (45), highlighting its potential association with IR in individuals with obesity (46). *CCL5* gene knockout significantly ameliorated high-fat diet (HFD)-induced inflammatory reactions in epididymal white adipose tissue and protected against the development of IR and obesity in mice fed HFD compared to control mice (47).

Low-grade chronic inflammation is a prominent characteristic of obesity in children and adults (41, 48). The presence of inflammation-associated cells in adipose tissue from subjects with obesity leads to a switch in macrophages phenotype from M2 (resident macrophages) to M1 (pathogenic macrophages), mainly through the activation of NF-kappa B signaling pathway, which is positively regulated by the generation of reactive oxygen species (ROS) and lipotoxicity (41, 42). ROS production plays a crucial role as a mediator of pro-inflammatory pathways since obesity and hyperglycemia-induced ROS production induce M1-like macrophages (49, 50). In addition, ROS production triggers the MAPK, STAT1, and NF-kappa B signaling pathways, thereby influencing macrophage differentiation (49, 50). STAT1 emerges as a pivotal player in the regulation of mitochondrial function, insulin sensitivity, and obesity-associated inflammation (51). In line with this, the skeletal muscle of mice fed HFD exhibited T cell polarization toward a pro-inflammatory TH1 phenotype, accompanied by increased STAT1 phosphorylation (52). Metabolomic and gene expression profiling analyses in *STAT1* knockout mice (*STAT1^a-KO^*) revealed improved mitochondrial function in subcutaneous adipose tissue (SAT) of obese mice compared to control mice (53). Moreover, reduced adipocyte size and SAT inflammation were observed in *STAT1^a-KO^* mice (53).

*GATA3* is a transcription factor that plays a key role in determining cell lineage and development of several cell types, including adipocytes (54). Notably, *GATA3* is recognized as a regulator of innate immunity as it induces macrophage polarization and infiltration (54). *GATA3* upregulation in the VAT of subjects with obesity increased IL-6 secretion, which in turn activated macrophage infiltration by upregulating monocyte chemoattractant protein-1 (MCP-1, encoded by the *CCL2* gene) (54). This cascade suppressed IL-10 release by VAT, leading to muscle inflammation and ultimately culminating in IR (54). The upregulation of *GATA3* in VAT and SAT of individuals with obesity is positively correlated with impaired adipogenesis (55, 56). *GATA3* is predominantly expressed in preadipocytes, and its upregulation in obesity hinders the ability of the adipose tissue to recruit new fat cells, resulting in ectopic fat deposition (56). Consistent with this, the suppression of *GATA3* has been shown to improve the adipogenesis process, restore insulin sensitivity, and reduce obesity-associated inflammation in patients with obesity (57).

The module analyses provide a systems-level perspective, revealing two highly interconnected modules associated with cellular processes, inflammation, and the regulation of lipid and glucose metabolism (58). Module 1 includes the following hypomethylated high-expression genes: *CBFA2T3*, *GATA3*, *LMO2*, and *TAL1*, with enriched KEGG pathways such as cellular cycle and cell senescence, TGF-β, JAK-STAT, HIF-1, and Wnt signaling pathways. The identification of senescence-associated genes within Module 1 adds an additional layer of complexity, linking cellular senescence to the pathogenesis of childhood obesity. Module 2 includes three genes (*EDNRB*, *GNA12*, and *PLCB1*), with enriched pathways related to inflammation and regulation of lipid and glucose metabolism. *EDNRB* and *GNA12* were identified as hypomethylated high-expression genes, while *PLCB1* was identified as a hypermethylated low-expression gene.

Senescent cells release various molecules that affect different cells and tissues in a paracrine manner, potentially leading to organ dysfunction (59, 60). In the adipose tissue, senescent cells can disrupt lipid storage and impair adipogenesis by increasing cell and nuclear size (59, 61). Moreover, hepatocytes under senescence conditions exhibit downregulated antioxidant and mitochondrial capacity, which correlates with impaired fatty acid metabolism and may contribute to lipid deposition in peripheral tissues, thereby exacerbating IR (59). Cellular senescence is also closely linked to inflammation (60). The upregulation of signaling pathways such as TGF (62, 63), JAK-STAT (64, 65), HIF-1 (66, 67), and Wnt (68, 69) may be interconnected, contributing to cell degradation and inflammation in various metabolic diseases, including obesity.

Adipocyte hyperplasia and hypertrophy increase the risk of obesity and its associated complications (70). The Wnt/β catenin signaling pathway plays a crucial role in regulating adipocyte cell expansion, differentiation, and cell death during infant growth (70, 71). This pathway also appears to regulate hypoxia (70) and is stimulated under obesogenic conditions in VAT, particularly with high caloric intake (70, 72), once it presents central roles in body fat distribution and carbohydrate and lipid metabolism (71, 72). The accumulation of fat, enhanced by the Wnt/β catenin pathway, triggers TLR-4 (toll-like receptor 4) responses in adipocytes facilitating the infiltration of macrophages into adipose tissue (73). This mechanism ultimately leads to the polarization of M2 macrophages (resident) into M1 phenotype (pathogenic), resulting in chronic inflammation through the release of pro-inflammatory factors (73). Despite the involvement of genes included in Modules 1 and 2 in different pathways, as described in Fig. 5C, we suggest that these molecules work together to achieve various functions within the childhood obesity-related network, contributing to the complexity of the disease.

Despite the promising insights, certain limitations must be acknowledged. First, the included datasets had relatively small sample sizes. While the DNA methylation datasets included both male and female children, the gene expression dataset included only one female. This limitation might restrict the generalizability of our results to women. Second, the DNA methylation datasets used blood samples, whereas the gene expression dataset used VAT biopsies. Consequently, our results could vary when considering other biological tissues. Third, the GSE25301 (containing methylome data) included patients with higher BMI (BMI = 39.0 ± 1.7) than the patients included in the other three datasets (GSE9624: BMI = 28.1 ± 0.7; GSE27860: BMI = 27.0 ± 1.0; and GSE57484: BMI = mean BMI z-score: 1.60 ± 0.2; all equivalent to patients with overweight). Thus, we cannot exclude the possibility that we might have not identified some MeDEGs strictly involved in patients with higher BMI. In addition, the absence of validation using VAT samples underscores the necessity for further experimental validations to confirm the *in-silico* findings. Furthermore, the datasets included in our comprehensive study did not contain both methylome and transcriptome data from the same individuals; therefore, the data originate from different cohorts, and our results should be interpreted with caution. Moreover, in the present study, methylome analyses were restricted to CpG islands within promoter regions. Consequently, the observed DNA methylation alterations are most likely indicative of either transcriptional activation or repression, contingent upon the magnitude and direction of methylation changes (25).

In conclusion, our study provides a comprehensive exploration of MeDEGs associated with childhood obesity, presenting potential candidate genes and pathways for further investigation. The identification of hub-bottleneck genes and functional modules contributes to understanding the complex networks putatively influencing childhood obesity. Experimental validations are essential to confirm these *in-silico* findings and pave the way for targeted interventions to mitigate the impact of childhood obesity on long-term health outcomes.

## Supplementary materials









## Declaration of interest

The authors declare that there is no conflict of interest that could be perceived as prejudicing the impartiality of the work reported.

## Funding

This study was partially funded by grants from Coordenação de Aperfeiçoamento de Pessoal de Nível Superior (CAPES), Conselho Nacional de Desenvolvimento Científico e Tecnológico (CNPq), Financiamento e Incentivo à Pesquisa at Hospital de Clínicas de Porto Alegre (FIPE-HCPA), and Postgraduation Program in Medical Sciences: Endocrinology at Universidade Federal do Rio Grande do Sul, Brazil. DC is a recipient of a scholarship from CNPq; FMP was a recipient of a scholarship from CAPES; and GCG was a recipient of a scholarship from HCPA (grant no. 23092.012897/2021-71).

## Author contribution statement

FMP, GCKD, GCG and TWK helped in conceptualization, methodology, formal analysis, investigation, writing of the original draft, writing of the review and editing and visualization. TSA helped in conceptualization, methodology, formal analysis, resources, writing of the review and editing, supervision and funding acquisition. DC helped in conceptualization, methodology, formal analysis, writing of the review and editing and supervision. This manuscript has been read and approved by all authors and all authors believe that this study represents honest work.
